# Carbon Threads Supercapacitors for Washable e-Textile Applications: Configurations and Electrochemical Performance

**DOI:** 10.1021/acsaenm.3c00723

**Published:** 2024-02-02

**Authors:** João Tiago Henriques, Catarina Cidade do Carmo, Ana Marques, Isabel M. M. Ferreira, Ana Catarina Baptista

**Affiliations:** † CENIMAT|i3N, Department of Materials Science, School of Science and Technology, NOVA University Lisbon, Caparica 2829-516, Portugal; ‡ Physics Department, Faculty of Sciences, University of Lisbon, Lisbon 1749-016, Portugal

**Keywords:** carbon electrodes, supercapacitors, electronic textiles, polypyrrole, washability

## Abstract

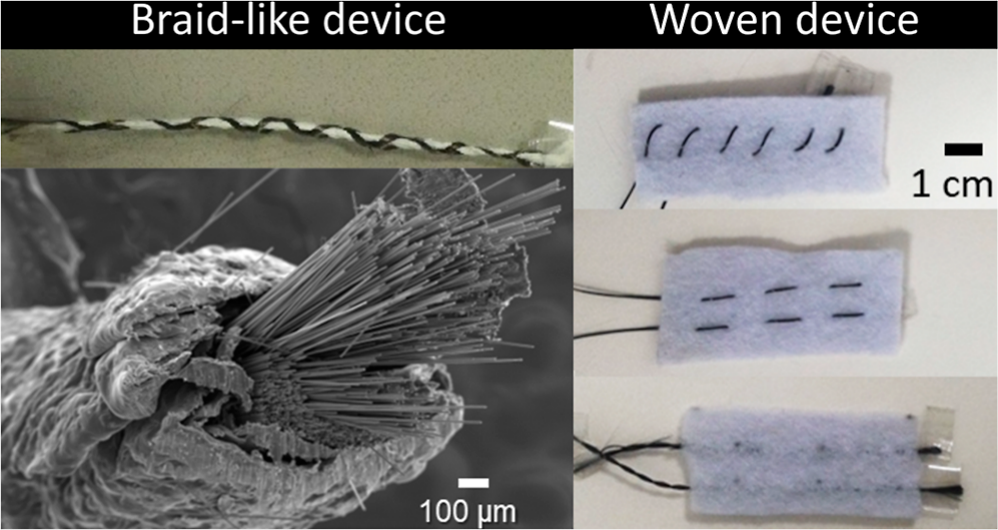

Technological solutions for emerging e-textiles are being sought to enable e-wear technology to be self-sustaining and lightweight. A rippling 1D carbon fiber capacitor design was made with commercial carbon threads as electrodes using simulated sweat solution as the electrolyte. This is particularly relevant for potential sports textile applications in which sweat could serve as an electrochemical energy source. An electrospun cellulose acetate fiber membrane and a commercially available felt were used as separators capable of soaking the electrolyte. These were tested in braided and woven electrode configurations, respectively. Functionalizing the carbon wires with polypyrrole (PPy) enhanced the surface area and significantly increased the specific capacity by approximately an order of magnitude (0.62 F/g). Cyclic voltammetry and charge–discharge tests confirmed the washability and durability of the devices for at least 1000 cycles.

## 1 Introduction

The integration of electronic gadgets into wearable items has been enhancing functionalities such as heartbeat, and body temperature monitoring, among other applications,^1−3^ in both occasional and more technical clothing, which are becoming increasingly sophisticated.^4^ Wearable electronic devices have evolved rapidly, but compatible power supplies are still in development. In response, one-dimensional (1D), two-dimensional (2D), and three-dimensional (3D) electric power supply structures have been researched and proposed as architectures for wearable electronics.^5−8^ A recent 1D device combined coaxial and twisted configurations, along with aligned nanotubes, anchored on a conductive substrate, for wearable energy harvesting devices, energy storage devices, and hybrid devices.^9−12^ 2D architectures, exploring 2D materials like graphene,^13^ transition metal oxides,^14^ black phosphorus,^15^ and others, leverage their planar electrical and thermal conductivity and flexibility.^4,16^ Sponge-like 3D structures benefit from the porous nature of some bulk materials to achieve flexible bulk devices when soaked in electrolyte, due to the high electrode surface area.^17,18^ 1D energy storage devices, such as fiber-shaped supercapacitors, are gaining importance due to their flexibility, low weight, and high compatibility with apparel manufacturing. However, wearable energy harvesting and storage devices still face challenges such as limited mechanical durability, poor electrical conductivity, and shorter life compared to traditional ones. Much research has been conducted to address these potential weaknesses and develop market-ready products. In a previous work, the authors demonstrated the functionality of supercapacitors based on carbon thread electrodes with cellulose acetate electrospun membrane for the dielectric layer, and sweat-like electrolyte,^19^ achieving a specific capacity of 2.3 F g^–1^, 386.5 mW h kg^–1^, and 46.4 kW kg^–1^ energy-density (*E*_d_) and power-density (*P*_d_), respectively. The present work aims to demonstrate the applicability of 1D and 2D supercapacitors on textiles using different configurations and further demonstrate the effect of wash cleaning on their electrochemical performance.

## 2 Experimental Section

### 2.1 Preparation of Supercapacitors with 1D and 2D Configurations

**Figure 1:**
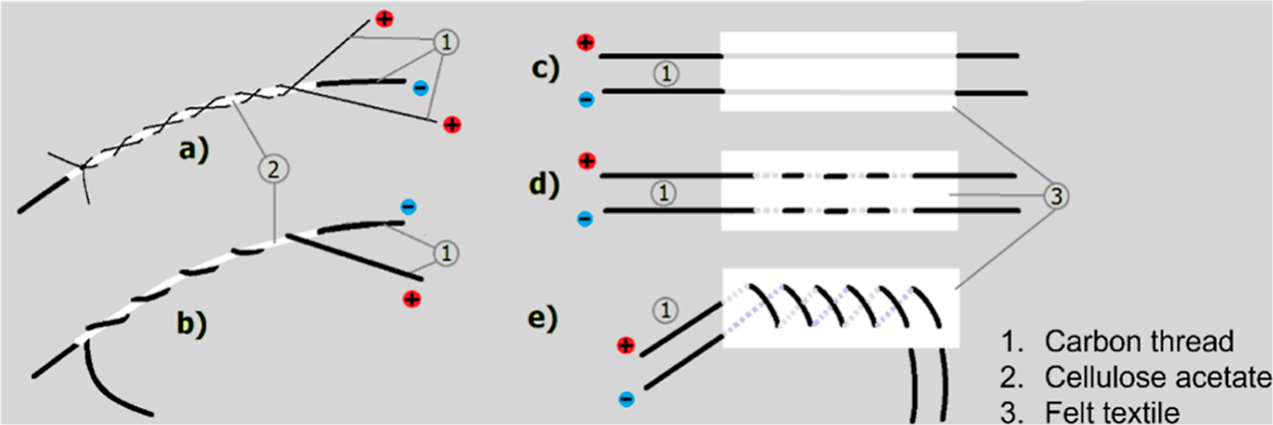
Schematics of the tested 1D and 2D device configurations. Braid-like and twisted-like configurations represented on (a,b), respectively. 2D internal, parallel, and crossed electrodes configurations depicted on (c–e), respectively.

The methodology for preparing 1D devices has been detailed in previous work, which includes the preparation of electrospun nano fibers, the functionalization of carbon yarns with polypyrrole PPy, and the use of simulated sweat solution (SSS) as an electrolyte.^19^ Commercial carbon threads (from TENAX, 218 Ω/m) each 10 cm in length, whether uncoated or coated with PPy electrodes, were manually twisted after being covered with a dielectric layer of cellulose acetate (CA) electrospun fibers. The 1D architecture consists of two fiber-shaped configurations: a braid configuration and twisted configuration, as illustrated in Figure 1a,b, respectively. The 2D configuration was tested in three different electrode configurations. Two carbon yarns (acting as electrodes) were manually woven into a piece of hydrophilic felt fabric, as depicted in Figure 1c–e, featuring internal electrodes, parallel electrodes, and crossed electrodes, respectively. PPy-coated yarns, each 7 cm long, were woven into a hydrophilic cotton fabric measuring 1.5 × 3 cm^2^.

### 2.2 Characterization

The electrical characterization of devices was performed using cyclic-voltammetry (CV) and cyclic-charge–discharge (CCD) tests on a potentiostat (Gamry Instruments-Reference 3000). CV was carried out with a sweep voltage range of 2 V (from −1 to 1 V) across five different scan-rates (10, 20, 50, 100, and 200 mV/s). Regarding the CCD experiments, a charge voltage from 0 to 0.5 V was followed by discharge voltage from 0.5 to 0 V, using five different charge–discharge currents (5, 10, 15, 20, and 25 μA). The accumulated charge and sweep voltage window were assessed from CV curves using Gamry Echem Analyst software. A single electrode mass was used for this calculation.

The electrolyte employed for testing the devices was a simulated sweat solution (SSS). This aqueous solution comprised sodium chloride (Sigma-Aldrich, 99.5%), sodium phosphate monobasic (Fluka analytical, 90%), and l-histidine (Sigma-Aldrich, 99%) in concentrations of 0.5, 0.22, and 0.05 % wt/V, respectively, as per ref (20) and according to ISO105-E04:2013. All devices were tested in electrolyte-saturated condition. For the 1D configuration, 45 μL of SSS was used to soak the electrospun membranes; whereas for the woven 2D configuration, 500 μL was applied to the felt fabric.

Electrodes surface morphology was examined using scanning electron microscopy (SEM) (model Hitachi S2400) with a gold–palladium sample coating.

The washing resistance of the supercapacitor was evaluated by performing CV measurements before the first washing cycle for all samples and after five washing cycles, using a 100 mV/s scan-rate, with a 1 to −1 V sweep voltage range across 10 cycles. For each washing cycle the device was immersed in a 100 mL aqueous solution of tap water and laundry soap (200 μL) for 15 min under magnetic stirring (200 rpm), simulating a real washing cycle. A tea bag containing the device was used to prevent the threads from becoming entangled around the magnet. After the washing cycle, the device was removed from the tea bag, any residual laundry soap was rinsed off with tap water, and it was dried on a heating plate at 40 °C for 20 min. Immediately following this process, CV measurements were performed.

## 3 Results and Discussion

### 3.1 1D Configuration

**Figure 2:**
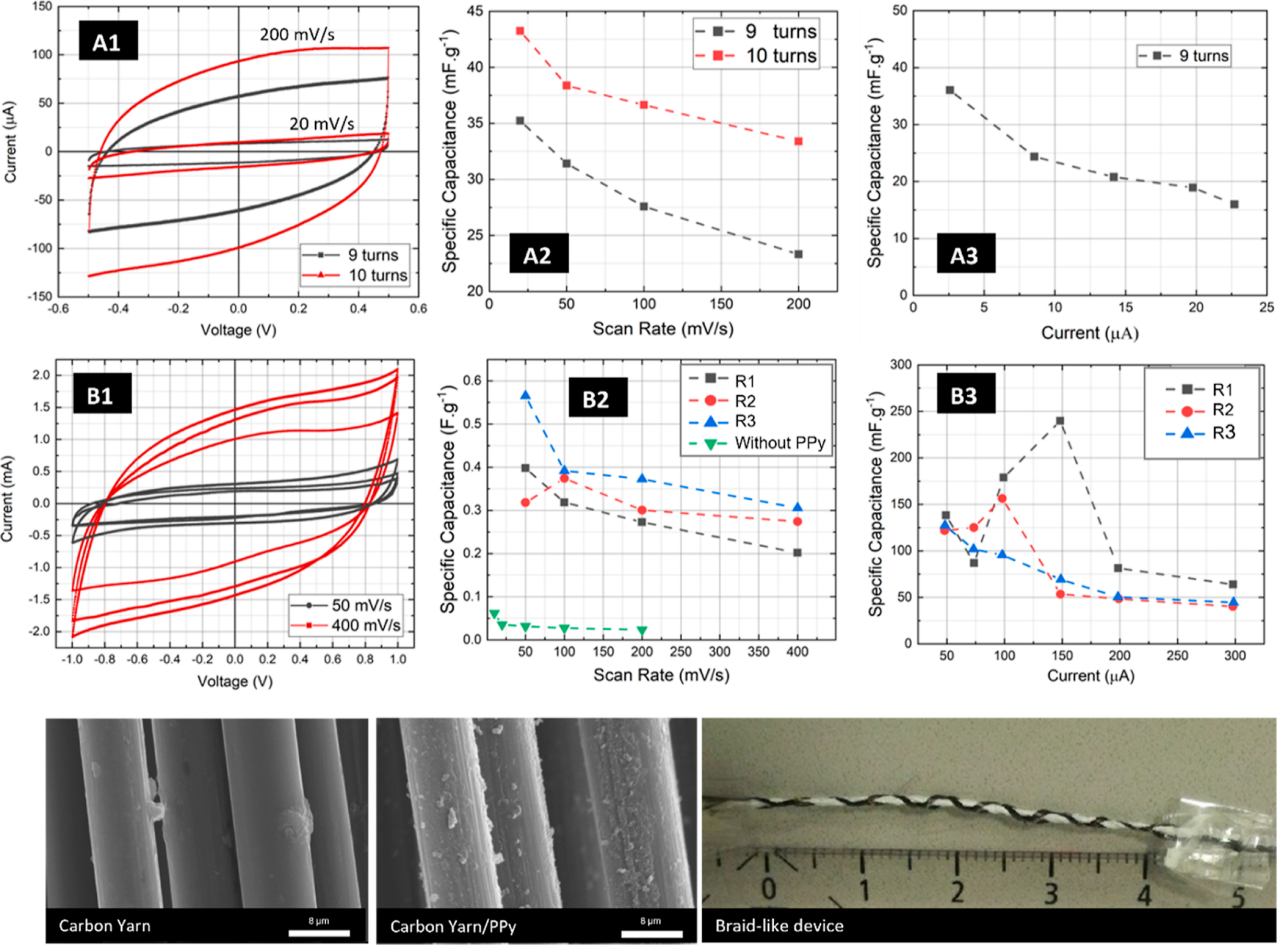
Influence of the number of turns (A) and replicas (B) of a braid-like 1D configuration PPy functionalized carbon fiber devices on cyclic voltammetry (A1,B1); specific capacitance vs scan rate (A2,B2); and specific capacitance vs discharge current (A3,B3). SEM images of carbon threads and carbon threads with PPy, and a photograph of a C|CA|C braid-like device is depicted in the bottom figure, respectively.

Figure 2A,B display the cyclic voltammetry (CV) and the specific capacity (SC) versus the scan rate along with charge–discharge (CD) measurements for different charge current of 1D braid-like devices. These devices have 9 and 10 turns of the outer electrode intertwining the inner electrode, with and without surface functionalization with PPy.

The quasi rectangular shape of the CV curves in Figure 2A1,B1 is typical of the formation of an electrical double layer. The decrease in specific capacity with increasing scan rate (see in Figure 2A2 and B2) is attributed to the reduced time available for the ions to diffuse and accumulate on the electrodes.^19,21^

The number of interweaving turns affects the specific capacity as the contact area of the external electrode increases. Notably, a significant increase in current is observed for carbon threads functionalized with PPy (Figure 2A2). This is due to an increase in surface area facilitated by the PPy molecules.^19^

Despite the carbon thread comprising multiple wires with approximately a 10 μm diameter, which contributes to a substantial surface area for the electrodes, the specific capacity of porous carbon is constrained, primarily due to limited contributions from specific surface capacitance values.^22^ The introduction of PPy molecules onto the surface of carbon threads, as observed in SEM images (by comparing carbon threads with and without PPy), enhances the surface roughness and, consequently, the surface area of fibers. The increased surface area leads to an enhancement of the electrode’s specific capacitance, and thereby improving the electrochemical performance of the device.^23^ Additional optical microscopy images and SEM cross-sectional views of uncoated and coated carbon yarns can be seen in the Supporting Information (Figure S1a–d). Supporting Information Figure S1e shows a cross-sectional view of a 1D configuration device. The existence of polypyrrole (PPy) was previously established through Raman spectroscopy, in which the identification of distinctive vibrational modes, including the bipolaron ring deformation, polaron symmetric C–H in-plane bending, ring stretching, and C=C stretching vibrations of PPy, were assigned to the respective wavenumbers of 930, 980, 1048, 1365, and 1581 cm^–1^.^19^

The reproducibility of the PPy layer was evaluated by creating three replica devices (Figure 2B2,B3). Despite the apparent significant variability in the results, which is largely due to the manual nature of the process, it is reproducible within a certain range of values. Earlier studies have also taken into account the variability of the electrolyte impregnated in the membrane, which is controlled but tends to gradually dry out over time,^19^ further contributing to this variability.

Overall, devices functionalized with PPy demonstrate a specific capacitance nearly an order of magnitude higher than those without functionalization (Figure 2B2). The results were obtained with 45 μL of electrolyte in various charge–discharge tests. Although the devices are reproducible, the deviation in the trend observed at 100 and 150 μA for specific capacitance could be related to an oversaturation of the cellulose fibers with simulated sweat solution (SSS).

### 3.2 2D Configuration

**Figure 3:**
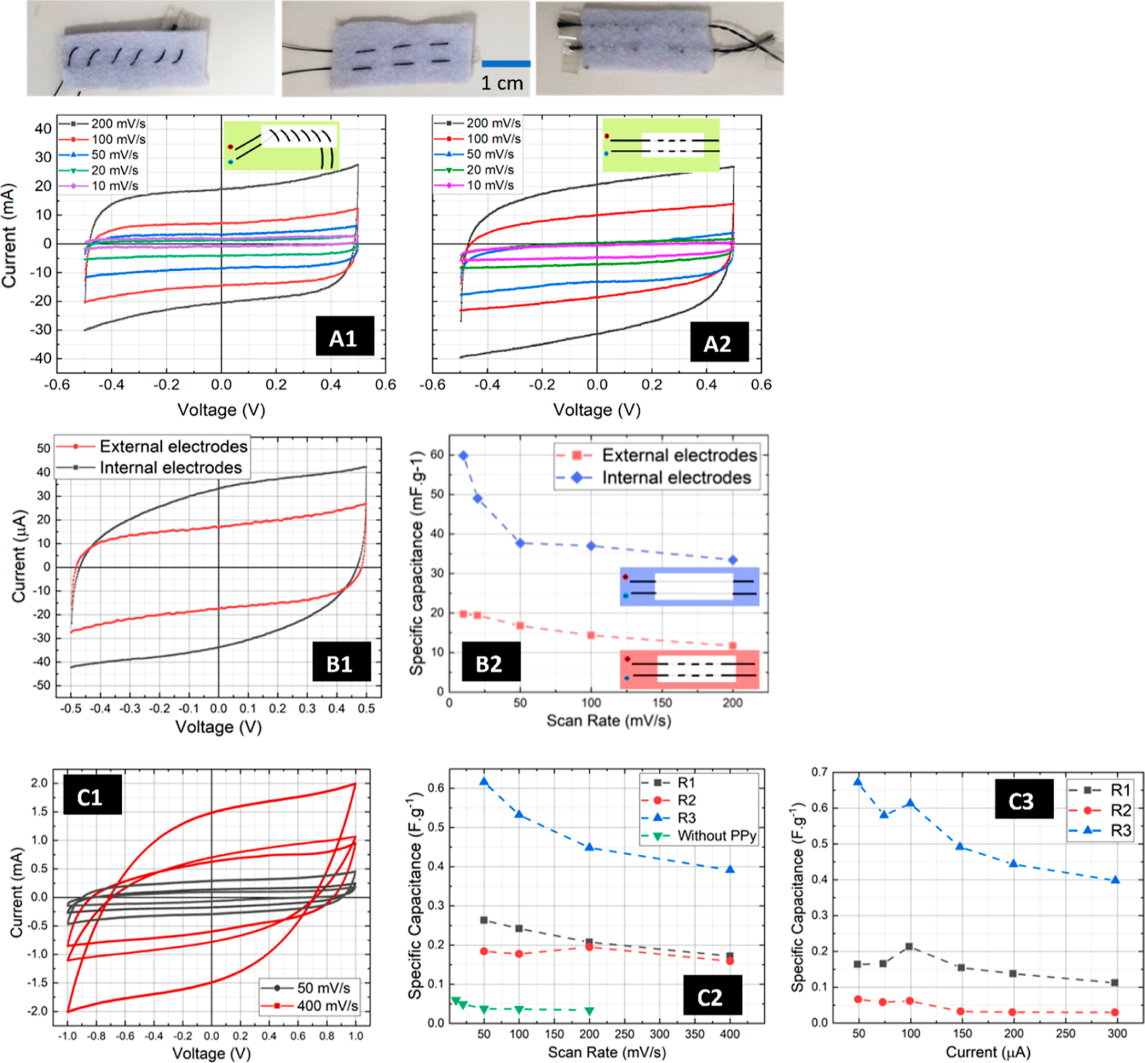
Photograph of the tested devices. (A1,A2) CV curves of cross and parallel woven configurations obtained at several scan rates, respectively. (B1) CV curves (9th curve of the 10 cycle CV experiment for 100 mV/s scan-rate) of parallel and internal woven configuration; (B2) specific capacitance versus scan rate for both internal and external yarn electrodes; (C1) CV results from three similar 2D woven devices (internal electrodes) with PPy functionalized electrodes, 0.3 cm apart; (C2) SC versus scan rate for the tested replicas; and (C3) SC vs current for the same devices.

In contrast to our group’s previous work,^19^ a new approach of intertwining the external electrode was tested, but the contact area remains limited. Consequently, a new 2D electrode configuration was attempted consisting of two carbon threads (electrodes) woven in a felt fabric. This configuration was chosen for its ease of replication in industrial applications. The different electrode designs tested included cross, parallel, and internal electrodes (Figure 1c–e). The cyclic voltammetry (CV) results indicated that the cross and parallel configurations performed similarly (Figure 3A1,A2), leading to the selection of the parallel design for comparison with the internal design (Figure 1c). The specific capacitance of the internal electrodes is almost double that of external electrodes in devices with pristine carbon threads (Figure 3B1,B2). The electrodes are separated by approximately 3 mm in both configurations. Therefore, the internal configuration was chosen for CV experiments with PPy-functionalized electrodes. The potential removal of some PPy coating during the insertion of the thread into the felt fabric could reduce performance, necessitating special care when weaving the fibers into the fabric. This effect is evident in Figure 3C1–C3, where two replicas show similar specific capacitance values, albeit lower than those of the braid-like configuration. This may be due to partial PPy detachment from the carbon yarn surface while inserting it into the felt, as the presence of a black residual powder was observed. In contrast, one of the replicas appears to have been inserted without damaging the PPy layer. The specific capacitance values are comparable to those of the braid-like configuration, which is expected considering the similarity in functionalization process for both configurations and the fact that the mechanism of charge accumulation is directly related with the surface area available to retain the charges.

### 3.3 Cyclic Charge–Discharge Durability

**Figure 4:**
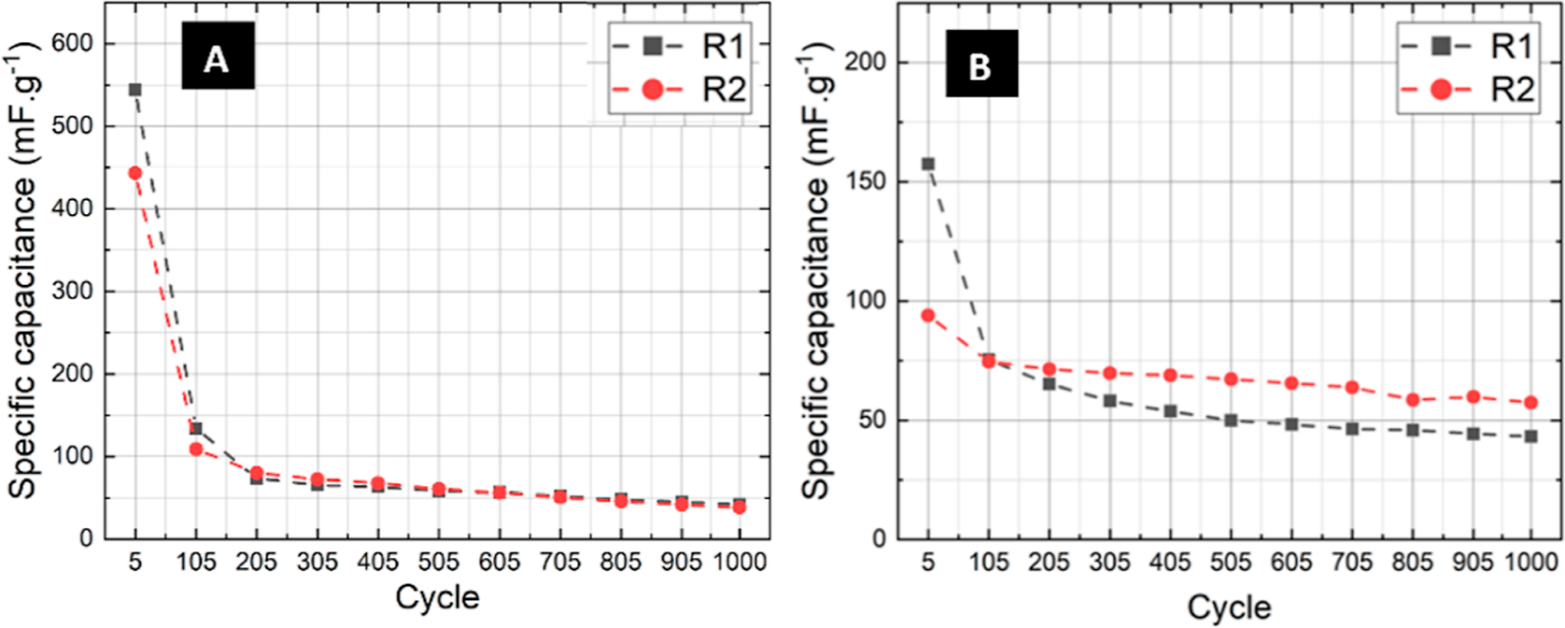
Specific capacitance vs number of charge–discharge cycles for two replicas of braidlike (A) and woven (B) devices with internal electrodes, at a constant current of 100 μA.

The cyclic stability of supercapacitors is a critical factor for the device success of reusable e-textile applications. A test involving 1000 cyclic charge–discharge was conducted to analyze the endurance of the supercapacitor, using 45 μL of electrolyte. In our previous work, the stability for 1000 cycles was measured with the device immersed in the SSS electrolyte. However, the objective of this study was to replicate a real-life and practical scenario where the intermittent presence of electrolytes from the user’s sweat would occur, subsequently followed by a natural drying process. Figure 4 illustrates a decrease in specific capacitance up to the 105th cycle, after which it stabilizes in the subsequent cycles. This behavior is attributed to the evaporation of water, leaving the salts within the membrane. However, due to the reduced diffusivity of ions, the device stabilizes with a specific capacitance of about 50 mF/g.

### 3.4 Washability Study

**Figure 5:**
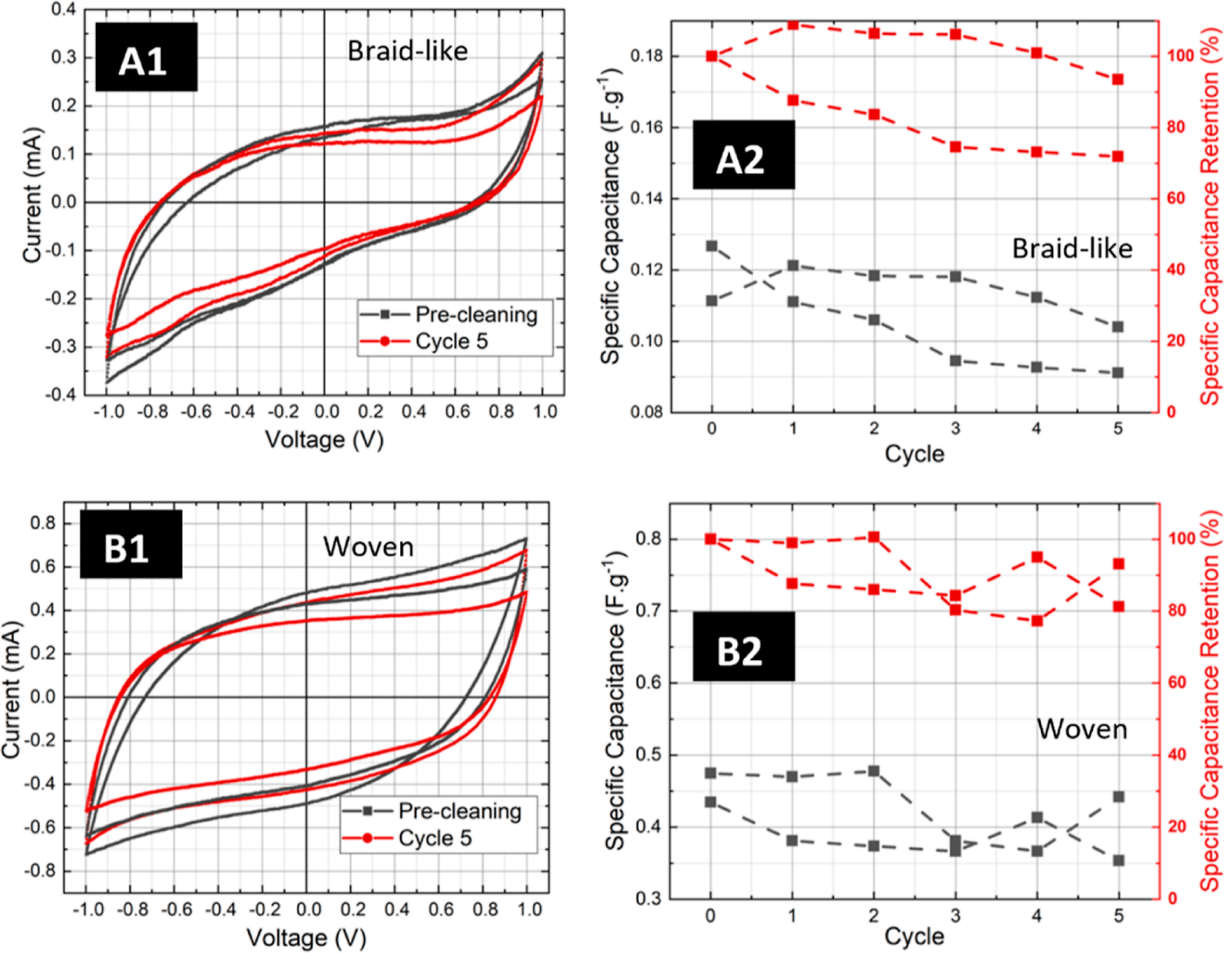
Influence of washing cycles on the cyclic voltammetry, specific capacitance, and specific capacitance retention of two-replicas: (A) 1D braid-like devices and B woven devices. (A1,B1) represented the ninth CV curve of 10 of each experiment with a scan rate of 100 mV/s before starting the washing experiment, and after every washing from a total of five cycles, sweep voltage window from −1 to 1 V. (A2,B2) Displays the washing cycles impact on the specific capacitance and specific capacitance retention of two replica devices.

The utilization of sweat as an electrolyte not only enhances biocompatibility but also imparts a notably eco-friendly aspect to the device, circumventing the need for strong acids, bases, and other aggressive electrolytes. Since the device’s electrolyte is not encapsulated, its washability becomes a crucial factor for its application in reusable garments. A preliminary study on washability of braid-like devices without PPy functionalization has revealed promising stability for e-textile applications. However, it has been established that PPy functionalization significantly improves the device’s specific capacitance. Consequently, understanding the impact of washing cycles on the PPy coating and therefore on the specific capacitance is of paramount importance. The effects of five washing cycles on CV, SC, and SC retention of two replica samples are depicted in Figure 5.

Two devices with braid-like and woven configurations underwent five washing cycles of 15 min each, following the protocol described in the Experimental Details. The specific capacitance results indicate that the tested devices were able to retrain most of their original performance throughout five cycles. Some variations were observed, which could be attributed to experimental changes combined with minor electrode movement during the washing cycles, resulting in a slight alteration of the electrode’s active area. Furthermore, when the CV before washing was compared with that after the final washing cycle, there was an average retention of over 80%.

The feasibility of using sweat as an electrolyte, coupled with the devices’ capacity to withstand washing tests, represents significant progress in the field of e-textile. For effective operation, practical electronic devices require a specific level of power and output voltage that cannot be achieved by using a single supercapacitor. To enable electronic devices to function, two or more supercapacitors must be connected in either a series or parallel configuration to increase the output voltage and power, respectively. Table 1 compares the devices developed in this work to others reported in the literature, particularly focusing on washability studies.

**Table 1:** Main Characteristics of Capacitors Made with Carbon-based Electrodes and Fibers/Textile That Include Washability

capacitor structure	capacitance	electrolyte	retention	robustness
carbon ink/Ag-blend fabric^24^	36 mF/cm^2^	LiCl/PVA	90% (30k cycles)	flexible (bending radius from 173 to 23 mm). washable (10 times) with capacitance retention >80%
carbon fibres + PEDOT:PSS (3 × 3 cm^2^)^25^	∼ 60 mF/cm^2^	H_3_PO_4_/PVA	70% (6k cycles)	stretchable (bending angle of 180°). washable (five times)^a^
graphene ink/poly cotton^26^	2.7 mF/cm^2^	H_2_SO_4_/PVA	98% (15k cycles)	stretchable (bending angle of 180°). washable (5 times)
MoO_3_ NFs + CNT + PDMS (prewashed)^27^	28 mF/cm^2^	KOH/PVA	50% (6k cycles)	stretchable (100 cycles at ε = 120%). washable (10 times)
CY-PPy/AC/CY-PPy braid-like^19^	6 mF/cm^2^	SSS	100% (1k cycles)^b^	stretchable (bending angle of 180°). no washing tests
CY-PPy/AC/CY-PPy braid-like (this work)	5 mF/cm^2^	SSS	c	washable >70% retention after five cycles
CY-PPy/AC/CY-PPy woven (this work)	23 mF/cm^2^	SSS	c	washable >80% retention after five cycles

a After encapsulation with a flexible silicon rubber.

b With electrolyte replacement during test.

c Retention is not calculated since electrolyte was not replaced/added during test.

The table compares the properties of devices documented in the literature that are similar to those studied here and includes washability tests. Apart from the Li-based electrolyte, the SSS electrolyte used in our study is akin to poly(vinyl alcohol) (PVA) with salts, but our findings indicate a higher capacity postwashing. The distinction in results between braid and woven type devices lies in the fact that the surface area (2π*rh*) was calculated for both, assuming an average fiber diameter of 400 μm and length of 7 cm. While this is a reasonable approximation for the fabric configuration where the fiber is fully wrapped with electrolyte-impregnated fabric, in the braid type configuration, the fiber’s contact area with electrolyte is less than a quarter of this value. Taking this into account, we achieved values of about 23 mF/cm^2^, which aligns with those obtained for the fabric configuration and is more realistic since the fibers undergo the same functionalization with PPy.

## 4 Conclusions

This work explores the electrochemical behavior of carbon thread 1D and 2D supercapacitor design configurations. In the braid-like 1D configuration, an average specific capacitance of 0.57 F/g and mechanical stability were achieved. However, the low contact area of the outer electrode limited the enhancement of this value. Therefore, a new 2D electrode configuration approach consisting of two carbon threads (electrodes) woven into a felt fabric was explored. This revealed both mechanical and electrochemical stability, with a supercapacitor achieving a specific capacitance of 0.62 F/g. Woven configurations also demonstrated the ability to retain up to 80% of their initial specific capacitance at the end of the fifth cycle, contrasting with the braid-like configuration, which only retained up to 71% of its initial specific capacitance. Additionally, both device types exhibited good resistance to washing cycles, maintaining charge retention above 80% after the fifth washing cycle.

These types of supercapacitors could show promise in providing power for electronic devices designed for signaling purposes, such as LEDs, emergency communication systems, and similar devices, while keeping their wearability, lightweight, and flexible characteristics.

## Supporting Information Available

Additional optical and SEM images of coated and uncoated carbon yarns and cross-sectional view of the 1D device configuration (PDF)
