# Supplementary material for: Carbon Threads Supercapacitors for Washable e-Textile Applications: Configurations and Electrochemical Performance
Source: ACS Appl Eng Mater. 2024 Feb 2;2(2):415–21. doi: 10.1021/acsaenm.3c00723 (PMC10897876; doi:10.1021/acsaenm.3c00723)
Supplement: Supplementary file 1 — em3c00723_si_001.pdf [file em3c00723_si_001.pdf]

Supporting Information

Carbon threads supercapacitors for washable e-textile applications: configurations and electrochemical performance

João Tiago Henriques[1], Catarina Cidade do Carmo[1], Ana Marques[1,2], Isabel M. M. Ferreira[1*] and Ana Catarina Baptista[1*]

[1]CENIMAT|i3N, Department of Materials Science, School of Science and Technology, NOVA University Lisbon, Caparica, Portugal

[2]Physics Department, Faculty of Sciences, University of Lisbon, 1749-016 Lisbon, Portugal

*Corresponding author: imf@fct.unl.pt (Isabel M. M. Ferreira) and anacbaptista@fct.unl.pt (Ana C. Baptista)

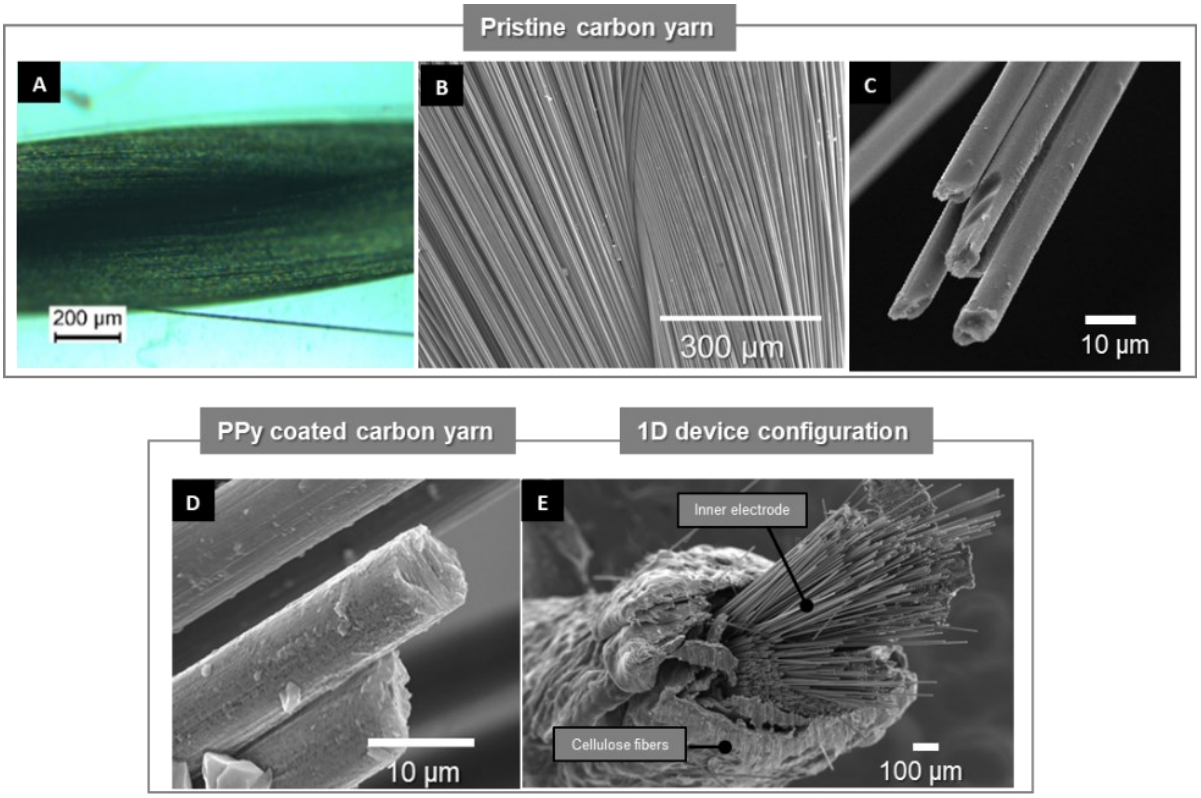

Figure SI1- (A) Optical microscopy image and (B) SEM image of pristine carbon yarn and cross-sectional views of the multifilament that composed the carbon yarn (C) pristine and (D) coated with PPy. (E) presents the cross-sectional view of a 1D device using a carbon yarn as the inner electrode and cellulose electrospun fibers as separator.
